# The Critical Period Hypothesis in Second Language Acquisition: A Statistical Critique and a Reanalysis

**DOI:** 10.1371/journal.pone.0069172

**Published:** 2013-07-25

**Authors:** Jan Vanhove

**Affiliations:** Department of Multilingualism, University of Fribourg, Fribourg, Switzerland; UCLA, United States of America

## Abstract

In second language acquisition research, the critical period hypothesis (cph) holds that the function between learners' age and their susceptibility to second language input is non-linear. This paper revisits the indistinctness found in the literature with regard to this hypothesis's scope and predictions. Even when its scope is clearly delineated and its predictions are spelt out, however, empirical studies–with few exceptions–use analytical (statistical) tools that are irrelevant with respect to the predictions made. This paper discusses statistical fallacies common in cph research and illustrates an alternative analytical method (piecewise regression) by means of a reanalysis of two datasets from a 2010 paper purporting to have found cross-linguistic evidence in favour of the cph. This reanalysis reveals that the specific age patterns predicted by the cph are not cross-linguistically robust. Applying the principle of parsimony, it is concluded that age patterns in second language acquisition are not governed by a critical period. To conclude, this paper highlights the role of confirmation bias in the scientific enterprise and appeals to second language acquisition researchers to reanalyse their old datasets using the methods discussed in this paper. The data and R commands that were used for the reanalysis are provided as supplementary materials.

## Introduction

In the long term and in immersion contexts, second-language (L2) learners starting acquisition early in life – and staying exposed to input and thus learning over several years or decades – undisputedly tend to outperform later learners. Apart from being misinterpreted as an argument in favour of early foreign language instruction, which takes place in wholly different circumstances, this general age effect is also sometimes taken as evidence for a so-called ‘critical period’ (cp) for second-language acquisition (sla). Derived from biology, the cp concept was famously introduced into the field of language acquisition by Penfield and Roberts in 1959 [1] and was refined by Lenneberg eight years later [2]. Lenneberg argued that language acquisition needed to take place between age two and puberty – a period which he believed to coincide with the lateralisation process of the brain. (More recent neurological research suggests that different time frames exist for the lateralisation process of different language functions. Most, however, close before puberty [3].) However, Lenneberg mostly drew on findings pertaining to first language development in deaf children, feral children or children with serious cognitive impairments in order to back up his claims. For him, the critical period concept was concerned with the implicit “automatic acquisition” [2, p. 176] in immersion contexts and does not preclude the possibility of learning a foreign language after puberty, albeit with much conscious effort and typically less success.

sla research adopted the critical period hypothesis (cph) and applied it to second and foreign language learning, resulting in a host of studies. In its most general version, the cph for sla states that the ‘susceptibility’ or ‘sensitivity’ to language input varies as a function of age, with adult L2 learners being less susceptible to input than child L2 learners. Importantly, the age–susceptibility function is hypothesised to be non-linear. Moving beyond this general version, we find that the cph is conceptualised in a multitude of ways [4]. This state of affairs requires scholars to make explicit their theoretical stance and assumptions [5], but has the obvious downside that critical findings risk being mitigated as posing a problem to only one aspect of one particular conceptualisation of the cph, whereas other conceptualisations remain unscathed. This overall vagueness concerns two areas in particular, viz. the delineation of the cph's scope and the formulation of testable predictions. Delineating the scope and formulating falsifiable predictions are, needless to say, fundamental stages in the scientific evaluation of any hypothesis or theory, but the lack of scholarly consensus on these points seems to be particularly pronounced in the case of the cph. This article therefore first presents a brief overview of differing views on these two stages. Then, once the scope of their cph version has been duly identified and empirical data have been collected using solid methods, it is essential that researchers analyse the data patterns soundly in order to assess the predictions made and that they draw justifiable conclusions from the results. As I will argue in great detail, however, the statistical analysis of data patterns as well as their interpretation in cph research – and this includes both critical and supportive studies and overviews – leaves a great deal to be desired. Reanalysing data from a recent cph-supportive study, I illustrate some common statistical fallacies in cph research and demonstrate how one particular cph prediction can be evaluated.

### Delineating the scope of the critical period hypothesis

First, the age span for a putative critical period for language acquisition has been delimited in different ways in the literature [4]. Lenneberg's critical period stretched from two years of age to puberty (which he posits at about 14 years of age) [2], whereas other scholars have drawn the cutoff point at 12, 15, 16 or 18 years of age [6]. Unlike Lenneberg, most researchers today do not define a starting age for the critical period for language learning. Some, however, consider the possibility of the critical period (or a critical period for a specific language area, e.g. phonology) ending much earlier than puberty (e.g. age 9 years [1], or as early as 12 months in the case of phonology [7]).

Second, some vagueness remains as to the setting that is relevant to the cph. Does the critical period constrain implicit learning processes only, i.e. only the untutored language acquisition in immersion contexts or does it also apply to (at least partly) instructed learning? Most researchers agree on the former [8], but much research has included subjects who have had at least some instruction in the L2.

Third, there is no consensus on what the scope of the cp is as far as the areas of language that are concerned. Most researchers agree that a cp is most likely to constrain the acquisition of pronunciation and grammar and, consequently, these are the areas primarily looked into in studies on the cph [9]. Some researchers have also tried to define distinguishable cps for the different language areas of phonetics, morphology and syntax and even for lexis (see [10] for an overview).

Fourth and last, research into the cph has focused on ‘ultimate attainment’ (ua) or the ‘final’ state of L2 proficiency rather than on the rate of learning. From research into the rate of acquisition (e.g. [11]–[13]), it has become clear that the cph cannot hold for the rate variable. In fact, it has been observed that adult learners proceed faster than child learners at the beginning stages of L2 acquisition. Though theoretical reasons for excluding the rate can be posited (the initial faster rate of learning in adults may be the result of more conscious cognitive strategies rather than to less conscious implicit learning, for instance), rate of learning might from a different perspective also be considered an indicator of ‘susceptibility’ or ‘sensitivity’ to language input. Nevertheless, contemporary sla scholars generally seem to concur that ua and not rate of learning is the dependent variable of primary interest in cph research. These and further scope delineation problems relevant to cph research are discussed in more detail by, among others, Birdsong [9], DeKeyser and Larson-Hall [14], Long [10] and Muñoz and Singleton [6].

### Formulating testable hypotheses

Once the relevant cph's scope has satisfactorily been identified, clear and testable predictions need to be drawn from it. At this stage, the lack of consensus on what the consequences or the actual observable outcome of a cp would have to look like becomes evident. As touched upon earlier, cph research is interested in the end state or ‘ultimate attainment’ (ua) in L2 acquisition because this “determines the upper limits of L2 attainment” [9, p. 10]. The range of possible ultimate attainment states thus helps researchers to explore the potential maximum outcome of L2 proficiency before and after the putative critical period.

One strong prediction made by some cph exponents holds that post-cp learners cannot reach native-like L2 competences. Identifying a single native-like post-cp L2 learner would then suffice to falsify all cph s making this prediction. Assessing this prediction is difficult, however, since it is not clear what exactly constitutes sufficient nativelikeness, as illustrated by the discussion on the actual nativelikeness of highly accomplished L2 speakers [15], [16]. Indeed, there exists a real danger that, in a quest to vindicate the cph, scholars set the bar for L2 learners to match monolinguals increasingly higher – up to Swiftian extremes. Furthermore, the usefulness of comparing the linguistic performance in mono- and bilinguals has been called into question [6], [17], [18]. Put simply, the linguistic repertoires of mono- and bilinguals differ by definition and differences in the behavioural outcome will necessarily be found, if only one digs deep enough.

A second strong prediction made by cph proponents is that the function linking age of acquisition and ultimate attainment will not be linear throughout the whole lifespan. Before discussing how this function would have to look like in order for it to constitute cph-consistent evidence, I point out that the ultimate attainment variable can essentially be considered a cumulative measure dependent on the actual variable of interest in cph research, i.e. susceptibility to language input, as well as on such other factors like duration and intensity of learning (within and outside a putative cp) and possibly a number of other influencing factors. To elaborate, the behavioural outcome, i.e. ultimate attainment, can be assumed to be integrative to the susceptibility function, as Newport [19] correctly points out. Other things being equal, ultimate attainment will therefore decrease as susceptibility decreases. However, decreasing ultimate attainment levels in and by themselves represent no compelling evidence in favour of a cph. The form of the integrative curve must therefore be predicted clearly from the susceptibility function. Additionally, the age of acquisition–ultimate attainment function can take just about *any* form when other things are not equal, e.g. duration of learning (Does learning last up until time of testing or only for a more or less constant number of years or is it dependent on age itself?) or intensity of learning (Do learners always learn at their maximum susceptibility level or does this intensity vary as a function of age, duration, present attainment and motivation?). The integral of the susceptibility function could therefore be of virtually unlimited complexity and its parameters could be adjusted to fit any age of acquisition–ultimate attainment pattern. It seems therefore astonishing that the distinction between level of sensitivity to language input and level of ultimate attainment is rarely made in the literature. Implicitly or explicitly [20], the two are more or less equated and the same mathematical functions are expected to describe the two variables if observed across a range of starting ages of acquisition.

**Figure 1:**
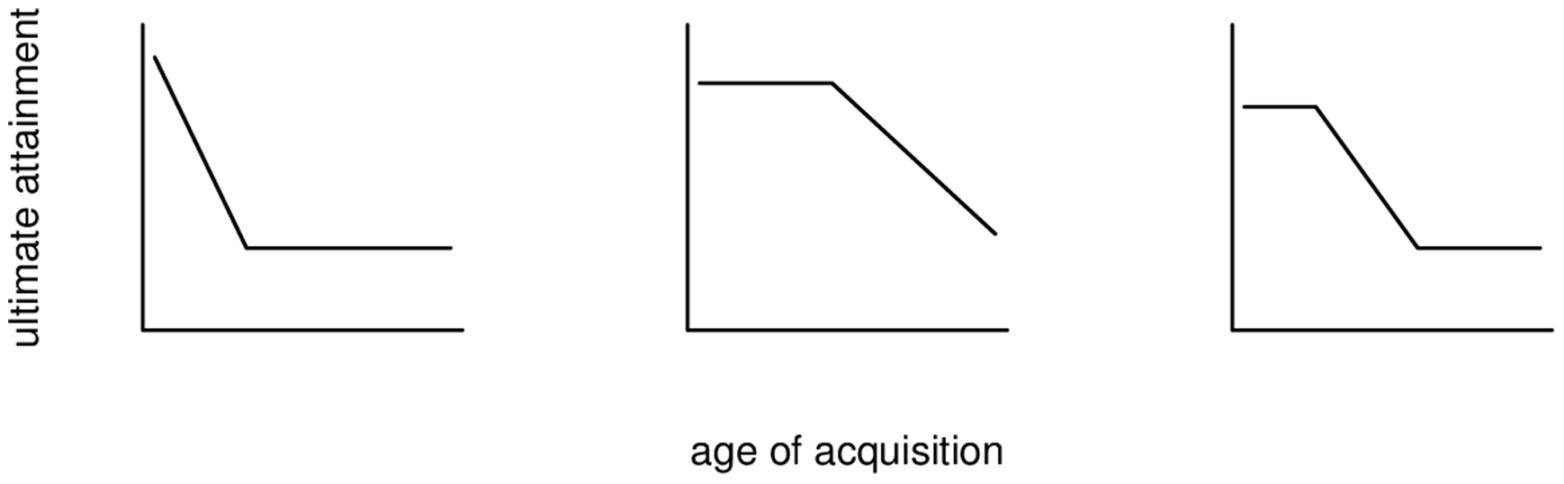
Three possible critical period effects. The graphs are based on based on Figure 2 in [9].

But even when the susceptibility and ultimate attainment variables are equated, there remains controversy as to what function linking age of onset of acquisition and ultimate attainment would actually constitute evidence for a critical period. Most scholars agree that not any kind of age effect constitutes such evidence. More specifically, the age of acquisition–ultimate attainment function would need to be different before and after the end of the cp [9]. According to Birdsong [9], three basic possible patterns proposed in the literature meet this condition. These patterns are presented in Figure 1. The first pattern describes a steep decline of the age of onset of acquisition (aoa)–ultimate attainment (ua) function up to the end of the cp and a practically non-existent age effect thereafter. Pattern 2 is an “unconventional, although often implicitly invoked” [9, p. 17] notion of the cp function which contains a period of peak attainment (or performance at ceiling), i.e. performance does not vary as a function of age, which is often referred to as a ‘window of opportunity’. This time span is followed by an unbounded decline in ua depending on aoa. Pattern 3 includes characteristics of patterns 1 and 2. At the beginning of the aoa range, performance is at ceiling. The next segment is a downward slope in the age function which ends when performance reaches its floor. Birdsong points out that all of these patterns have been reported in the literature. On closer inspection, however, he concludes that the most convincing function describing these age effects is a simple linear one. Hakuta et al. [21] sketch further theoretically possible predictions of the cph in which the mean performance drops drastically and/or the slope of the aoa–ua proficiency function changes at a certain point.

Although several patterns have been proposed in the literature, it bears pointing out that the most common explicit prediction corresponds to Birdsong's first pattern, as exemplified by the following crystal-clear statement by DeKeyser, one of the foremost cph proponents:

[A] strong negative correlation between age of acquisition and ultimate attainment throughout the lifespan (or even from birth through middle age), the only age effect documented in many earlier studies, is not evidence for a critical period…[T]he critical period concept implies a break in the AoA–proficiency function, i.e., an age (somewhat variable from individual to individual, of course, and therefore an age range in the aggregate) after which the decline of success rate in one or more areas of language is much less pronounced and/or clearly due to different reasons. [22, p. 445].

DeKeyser and before him among others Johnson and Newport [23] thus conceptualise only one possible pattern which would speak in favour of a critical period: a clear negative age effect before the end of the critical period and a much weaker (if any) negative correlation between age and ultimate attainment after it. This ‘flattened slope’ prediction has the virtue of being much more tangible than the ‘potential nativelikeness’ prediction: Testing it does not necessarily require comparing the L2-learners to a native control group and thus effectively comparing apples and oranges. Rather, L2-learners with different aoas can be compared amongst themselves without the need to categorise them by means of a native-speaker yardstick, the validity of which is inevitably going to be controversial [15]. In what follows, I will concern myself solely with the ‘flattened slope’ prediction, arguing that, despite its clarity of formulation, cph research has generally used analytical methods that are irrelevant for the purposes of actually testing it.

### Inferring non-linearities in critical period research: An overview

In this section, I present a non-exhaustive overview of studies that have either claimed to have found evidence relevant to the ‘flattened slope’ prediction or that have been cited by others in this context. These studies can be split up in three broad and partially overlapping categories. The first category consists of studies in which statistical tools to compare means or proportions, e.g. 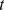
- and 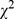-tests and anovas, were used. Studies in which the correlation coefficients of the aoa–ua relationship were compared between younger and older arrivals make up the second category. Lastly, studies in the third category used regression methods to address the ‘flattened slope’ prediction. I will demonstrate that the analyses used in the first two categories rest on statistical fallacies, rendering them useless for the purposes of addressing the ‘flattened slope’ prediction. Regression models, I argue, present the only valid alternative, provided they are fitted correctly and interpreted judiciously.

#### Group mean or proportion comparisons

The first broad category consists of studies in which the aoa continuum is discretised into bins (e.g. aoa 3–7, 8–10, 11–15 and 17–39 years in a study by Johnson and Newport [23]), whose ua scores or nativelikeness ratings are subsequently compared together and sometimes with those of native speakers using a series of 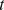
- or 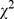-tests or an anova. Inferences about discontinuities in the aoa–ua function are then made on the basis of whether such comparisons reach significance or not. (To prevent any misunderstandings, note that the terms ‘discontinuity’ and ‘non-continuity’ are often used in cph research, even though the predicted patterns (see Figure 1) do not contain discontinuities in the mathematical sense. In mathematics, a discontinuity is a ‘jump’ in the function [24].) A fairly recent paper by Abrahamsson and Hyltenstam [15] is a case in point. The authors split up the aoa continuum into five bins (aoa 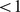
–5, 6–11, 12–17, 18–23 and 24–47 years), carried out an anova with pairwise post-hoc tests on nativelikeness ratings and inferred the presence of a critical point in adolescence on the basis thereof:

[T]he main differences can be found between the native group and all other groups – including the earliest learner group – and between the adolescence group and all other groups. However, neither the difference between the two childhood groups nor the one between the two adulthood groups reached significance, which indicates that the major changes in eventual perceived nativelikeness of L2 learners can be associated with adolescence. [15, p. 270].

Similar group comparisons aimed at investigating the effect of aoa on ua have been carried out by both cph advocates and sceptics (among whom Bialystok and Miller [25, pp. 136–139], Birdsong and Molis [26, p. 240], Flege [27, pp. 120–121], Flege et al. [28, pp. 85–86], Johnson [29, p. 229], Johnson and Newport [23, p. 78], McDonald [30, pp. 408–410] and Patowski [31, pp. 456–458]). To be clear, not all of these authors drew direct conclusions about the aoa–ua function on the basis of these groups comparisons, but their group comparisons have been *cited* as indicative of a cph-consistent non-continuous age effect, as exemplified by the following quote by DeKeyser [22]:

Where group comparisons are made, younger learners always do significantly better than the older learners. The behavioral evidence, then, suggests a non-continuous age effect with a “bend” in the AoA–proficiency function somewhere between ages 12 and 16. [22, p. 448].

The first problem with group comparisons like these and drawing inferences on the basis thereof is that they require that a continuous variable, aoa, be split up into discrete bins. More often than not, the boundaries between these bins are drawn in an arbitrary fashion, but what is more troublesome is the loss of information and statistical power that such discretisation entails (see [32] for the extreme case of dichotomisation). If we want to find out more about the relationship between aoa and ua, why throw away most of the aoa information and effectively reduce the ua data to group means and the variance in those groups?

Second, I strongly suspect that the underlying assumption when using 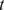
- and 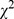-tests and anovas to infer the shape of the underlying aoa–ua function is one of the gravest fallacies in all of inferential statistics: the belief that non-significant test results indicate that the group means or proportions are essentially identical. To quote Schmidt, this notion is “the most devastating of all to the research enterprise” [33, p. 126]. Yet, judging by the snippet quoted above, Abrahamsson and Hyltenstam's reasoning seemed to be that the lack of a statistical difference between the childhood groups and between the adulthood groups indicates that these groups perform at roughly the same level, whereas the presence of a statistical difference between the adolescence group and all other groups indicates a steep drop in perceived nativelikeness. Such reasoning ignores the issue that when the default null hypothesis of no difference is adopted as or integrated into the research hypothesis, the statistical power of the tests, i.e. the probability of finding a statistically significant difference when the actual population means differ by a prespecified minimum effect size, should be substantially higher than what tends to be the case in the social sciences [34].

In order to illustrate the gravity of this problem, I computed the power that Abrahamsson and Hyltenstam would actually have had to detect a significant difference between their two childhood groups (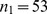, 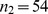) if the underlying population effect size had, in fact, been medium-sized (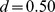, see [35]). These power computations were carried out with the pwr.t2n.test() function in the pwr package for R[36]. (R[37] is an open source program and programming language for statistical computing and can be downloaded freely from http://www.r-project.org/. All add-on packages used for the analyses in this paper can be installed from within r, see the ‘supporting information’ section. For a highly accessible introductory text to power analysis, see Cohen's *Power primer* [35].) It turned out that Abrahamsson and Hyltenstam's power was about 0.73 assuming a two-tailed 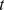-test with 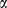 fixed at 0.05. While this is better than what is typically found in social science papers [34], it still means that in 27% of cases, even a medium-sized effect would have gone undetected. Since Abrahamsson and Hyltenstam used post-hoc tests that corrected the individual 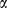-levels downwards to maintain the familywise Type I error rate, their actual power was even lower [38], [39]. To clarify, I am not arguing against maintaining the familywise 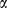 level; the point is merely that these power computations are generous. In the case of Johnson and Newport's oft-cited study, which claimed that participants with aoas between 3 and 7 years (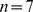) did not behave differently from native speakers (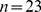) and on that basis surmised the presence of a non-continuity, this lack of power is even more pronounced at a mere 0.20, assuming a medium-sized effect size and a two-tailed test with 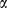 fixed at 0.05. This means that in a whopping 80% of cases a medium-sized effect would have gone undetected. Note that Sedlmeier and Gigerenzer [34] suggest that researchers have a power level of 0.95 before they accept null hypotheses, which is equivalent to the typical requirement of needing a 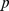-value lower than 0.05 before rejecting the null hypothesis in favour of a non-null research hypothesis, but which would require about 105 participants per group (assuming 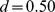).

Thus, within an ‘orthodox’ frequentist framework, group mean or proportion comparisons are fine for establishing that a difference *does* likely exist between two groups (though subject to a host of caveats, see [40]–[43] and many others), but using them to infer that a difference does *not* exist is highly suspect. The only reliable inference that they by themselves allow in cph research is that younger learners tend to outperform older learners in some domains of language (e.g. pronunciation and syntax), which all scholars implied in the debate essentially agree on. In sum, inferring the precise shape of a bivariate relationship using 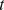-tests, anovas or 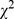-tests is at the very least cumbersome and prone to errors.

#### Comparison of correlation coefficients

The second broad category, which is not mutually exclusive with the first category, consists of studies that address the discontinuity hypothesis by computing and comparing correlation coefficients between aoa and ua for two or more aoa subgroups. In a sense, this approach represents an improvement over group mean or proportion comparisons as the aoa data are treated as a continuous variable. Nevertheless, this approach, too, rests on a fallacious assumption, namely that differences in correlation coefficients are indicative of differences in slopes. We suspect that the correlation-based approach dates back to Johnson and Newport's 1989 study [23], in which they split up their participants into two aoa-defined groups and found that ua as measured using a gjt correlated strongly and significantly in the early arrivals (age 3–15, 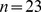, 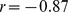) but not in the older arrivals (age 17–39, 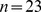, 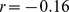). Johnson and Newport took this to suggest that “language learning ability slowly declines as the human matures and plateaus at a low level after puberty” [23, p. 90].

Correlation-based inferences about slope discontinuities have similarly explicitly been made by cph advocates and skeptics alike, e.g. Bialystok and Miller [25, pp. 136 and 140], DeKeyser and colleagues [22], [44] and Flege et al. [45, pp. 166 and 169]. Others did not explicitly infer the presence or absence of slope differences from the subset correlations they computed (among others Birdsong and Molis [26], DeKeyser [8], Flege et al. [28] and Johnson [29]), but their studies nevertheless featured in overviews discussing discontinuities [14], [22]. Indeed, the most recent overview draws a strong conclusion about the validity of the cph's ‘flattened slope’ prediction on the basis of these subset correlations:

In those studies where the two groups are described separately, the correlation is much higher for the younger than for the older group, except in Birdsong and Molis (2001) [ =  [26], JV], where there was a ceiling effect for the younger group. This global picture from more than a dozen studies provides support for the non-continuity of the decline in the AoA–proficiency function, which all researchers agree is a hallmark of a critical period phenomenon. [22, p. 448].

In Johnson and Newport's specific case [23], their correlation-based inference that ua levels off after puberty happened to be largely correct: the gjt scores are more or less randomly distributed around a near-horizontal trend line [26]. Ultimately, however, it rests on the fallacy of confusing correlation coefficients with slopes, which seriously calls into question conclusions such as DeKeyser's (cf. the quote above).

For clarity's sake, let's briefly review the difference between correlation coefficients and slopes. The slope of a function is defined as the increment with which and the direction in which the value on the 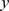-axis changes when the value on the 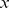-axis is increased by one increment. In a linear regression model of the form 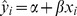, 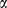 is the value of 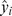 (i.e. the expected 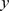-value according to the model) when 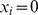, i.e. the intercept. The coefficient that 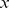 takes in this equation, 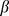, represents the slope of the regression function, i.e. it expresses how 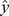 changes when 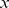 is increased by one increment. In principle, 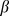 can take any value between negative and positive infinity.

**Figure 2:**
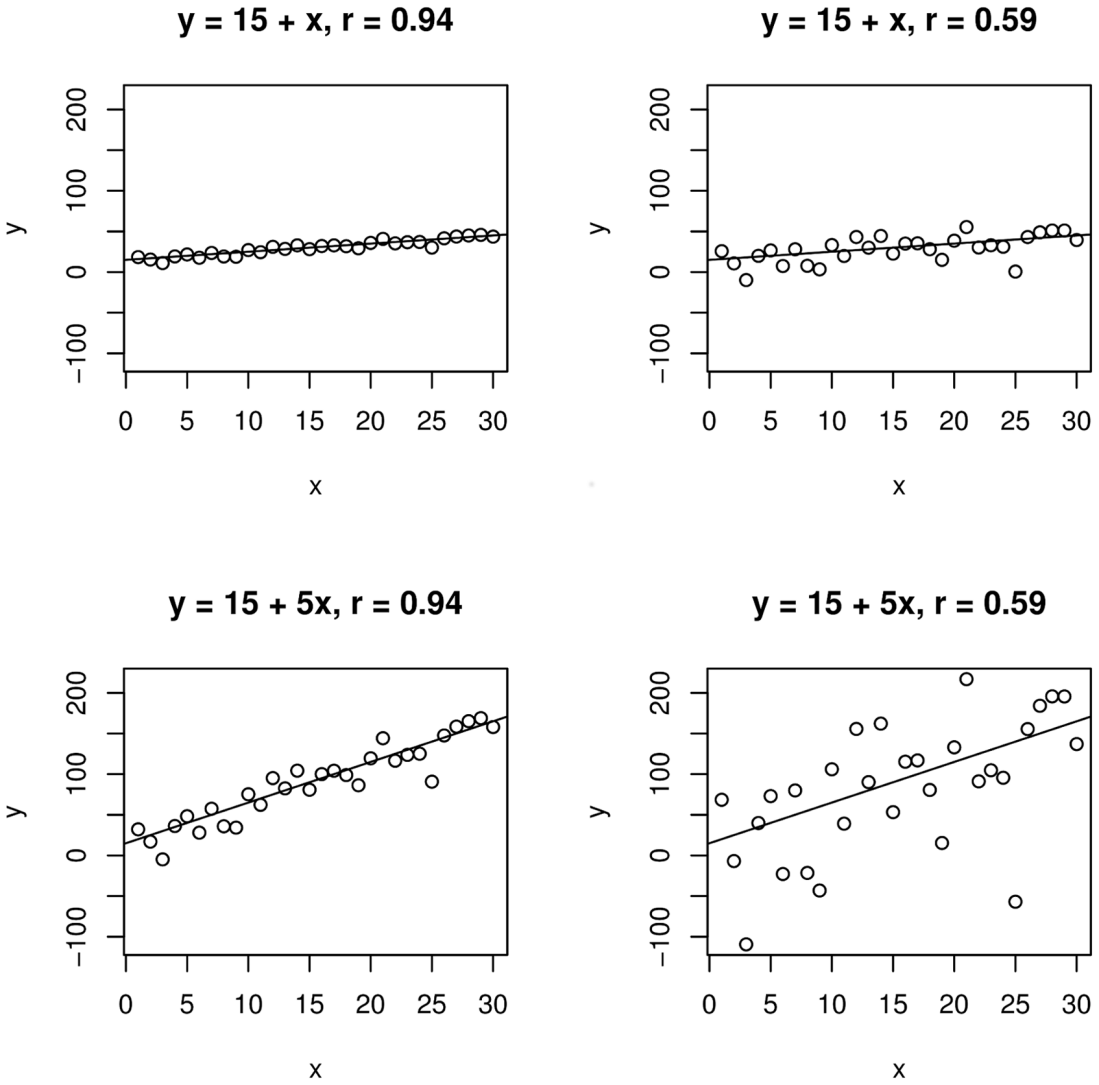
Illustration of the difference between correlation coefficients and slopes. Relationships on the same row were generated by the same underlying function (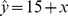 and 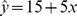, respectively) but are characterised by different correlation coefficients (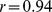 and 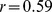, respectively). The inverse is true for relationships in the same column.

The Pearson correlation coefficient, 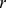, on the other hand, expresses the strength of the linear relationship between two variables. It is bound between 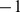 (perfect negative relationship) and 1 (perfect positive relationship). If 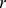 equals 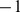 or 1, a straight line captures all the data points; the closer 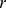 comes to zero, the farther from such a linear line the data points are scattered. In simple linear functions, 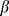 and 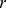 are linked to each other in that 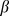 is 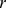 times the ratio of the sample standard deviations of the 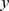
- and 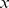-variables: 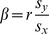. Crucially, however, the relationships between two pairs of variables can be characterised by the same functional regression form but still have radically different 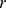 coefficients, and the other way around (see Figure 2).

What this boils down to is that a hypothesis concerning the slope of a function must be addressed by comparing 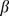 coefficients computed using regression techniques rather than by comparing correlation coefficients. But then why are the aoa–ua correlations typically weaker in the older arrivals than in the younger ones? Assuming, for the sake of the argument, that the slope of the aoa–ua function is identical in both groups (Eq. 1), we can substitute the 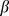 coefficients for the correlation coefficients times the ratio of the relevant sample standard deviations (Eq. 2).
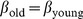
(1)

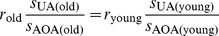
(2)


It can then straightforwardly be deduced that, other things equal, the aoa–ua correlation in the older group decreases as the ua variance in the older group increases relative to the ua variance in the younger group (Eq. 3).
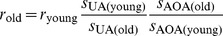
(3)


Lower correlation coefficients in older aoa groups may therefore be largely due to differences in ua variance, which have been reported in several studies [23], [26], [28], [29] (see [46] for additional references). Greater variability in ua with increasing age is likely due to factors other than age proper [47], such as the concomitant greater variability in exposure to literacy, degree of education, motivation and opportunity for language use, and by itself represents evidence neither in favour of nor against the cph.

#### Regression approaches

Having demonstrated that neither group mean or proportion comparisons nor correlation coefficient comparisons can directly address the ‘flattened slope’ prediction, I now turn to the studies in which regression models were computed with aoa as a predictor variable and ua as the outcome variable. Once again, this category of studies is not mutually exclusive with the two categories discussed above.

In a large-scale study using self-reports and approximate aoas derived from a sample of the 1990 U.S. Census, Stevens found that the probability with which immigrants from various countries stated that they spoke English ‘very well’ decreased curvilinearly as a function of aoa [48]. She noted that this development is similar to the pattern found by Johnson and Newport [23] but that it contains no indication of an “abruptly defined ‘critical’ or sensitive period in L2 learning” [48, p. 569]. However, she modelled the self-ratings using an ordinal logistic regression model in which the aoa variable was logarithmically transformed. Technically, this is perfectly fine, but one should be careful not to read too much into the non-linear curves found. In logistic models, the outcome variable itself is modelled linearly as a function of the predictor variables and is expressed in log-odds. In order to compute the corresponding probabilities, these log-odds are transformed using the logistic function. Consequently, even if the model is specified linearly, the predicted probabilities will not lie on a perfectly straight line when plotted as a function of any one continuous predictor variable. Similarly, when the predictor variable is first logarithmically transformed and then used to linearly predict an outcome variable, the function linking the predicted outcome variables and the untransformed predictor variable is necessarily non-linear. Thus, non-linearities follow naturally from Stevens's model specifications. Moreover, cph-consistent discontinuities in the aoa–ua function *cannot* be found using her model specifications as they did not contain any parameters allowing for this.

Using data similar to Stevens's, Bialystok and Hakuta found that the link between the self-rated English competences of Chinese- and Spanish-speaking immigrants and their aoa could be described by a straight line [49]. In contrast to Stevens, Bialystok and Hakuta used a regression-based method allowing for changes in the function's slope, viz. locally weighted scatterplot smoothing (lowess). Informally, lowess is a non-parametrical method that relies on an algorithm that fits the dependent variable for small parts of the range of the independent variable whilst guaranteeing that the overall curve does not contain sudden jumps (for technical details, see [50]). Hakuta et al. used an even larger sample from the same 1990 U.S. Census data on Chinese- and Spanish-speaking immigrants (2.3 million observations) [21]. Fitting lowess curves, no discontinuities in the aoa–ua slope could be detected. Moreover, the authors found that piecewise linear regression models, i.e. regression models containing a parameter that allows a sudden drop in the curve or a change of its slope, did not provide a better fit to the data than did an ordinary regression model without such a parameter.

Summarising, Bialystok and Hakuta and Hakuta et al. found no evidence supporting a cph account for the aoa–self-ratings relationship. The pertinence of these studies to the cph has, however, been questioned for a number of reasons. These concern (1) the exclusion of immigrants who reported that they only spoke English at home from the data set [51], (2) the possibility that the immigrants *believed* that second-language competence decreases monotonically as a function of age of learning and that the self-ratings are shaped by this belief [14], [52], (3) the coarseness of the aoa variable retrieved from the census [51], [52], and (4) the assumption that the self-ratings could be considered a continuous variable [51]. While I recognise the potential of all four points to obscure a cp effect in the aoa–ua function, I fail to grasp another point of Stevens's criticism of Hakuta et al.'s study. This point concerns the use of comparing simple linear regression fits to fits of piecewise linear regressions. She argues that since the aoa–proficiency relationship is negative when viewed over the whole lifespan, there is hardly any variance left to be explained by the breakpoints [51]. This is, of course, the whole point of the enterprise: parsimony dictates that if the breakpoints do not add sufficiently to the model fit, they should be left out! That said, the necessity of including a breakpoint in the model can be assessed by means other than the coefficient of determination (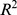), e.g. relative goodness-of-fit measures such as the Akaike Information Criterion [53] or the Bayesian Information Criterion [54] or 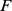-tests. Such measures can in principle indicate better model fits even if the increase in 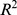 is minimal.

To my knowledge, regression models capable of highlighting non-linearities have only been modelled in two studies looking into the relationship between aoa and ua variables extracted using tasks rather than self-ratings. Flege et al. measured ua in English for 240 Korean participants using foreign-accent ratings and a grammaticality judgement task (gjt) [28]. They fitted both linear and cubic functions to the aoa–ua data. The cubic function explained somewhat more variance than did the linear function for the foreign-accent ratings (increase in 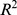: 1.9%), but follow-up analyses failed to find support for a non-linearity in puberty. A cubic function likewise explained somewhat more variance compared to a linear function for the gjt scores (increase in 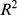: 1.2%), but this time follow-up analyses revealed a change in slope an aoa of about 12 years. In my opinion, however, Flege et al.'s follow-up analyses are not quite ideal as they entail fitting models on aoa-defined subsets and checking whether the cubic term still contributed significantly to the model fit in those subsets; I refer the reader to the original publication for details on this procedure. (Moreover, pinpointing the location of a slope change in a cubic function is mathematically speaking impossible: the function's slope changes continuously (expressed by the first derivative, which itself is a continuous quadratic function) as does the rate by which it changes (expressed by the second derivative, which is a continuous linear function). One could pinpoint the aoa at which the change in slope starts to slow down or speed up (i.e. the point at which the sign of the second derivative changes), but one should be aware that one is dealing with a continuous phenomenon.)

Instead, I prefer the analytical approach used by Birdsong and Molis, who, like Hakuta et al., fitted piecewise linear regression models and checked whether the breakpoint parameter contributed enough to the model to offset the resultant loss of parsimony [26]. Birdsong and Molis's study was a replication of Johnson and Newport's but used Spanish L1 speakers (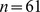) rather than Korean- and Chinese-speaking participants. These authors found a breakpoint in the aoa–ua slope that contributed significantly to the model fit, but this breakpoint was located at aoa 27.5 years – well beyond a putative critical period. Reanalysing Johnson and Newport's data, the authors further found that a breakpoint could improve the model fit for this data set, too. This time, however, the breakpoint was located at aoa 18 years. Importantly, the breakpoints had different functions in the two data sets: whereas it marked the beginning of a flatter part of the curve in the Johnson and Newport data set (as in the left panel of Figure 1), it actually marked the onset of a *steeper* part of the curve in the Birdsong and Molis study (as in the middle panel of Figure 1). In other words, the age effect in ua actually became more pronounced for the older arrivals. (Birdsong and Molis did not mention by how much 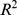 increased when breakpoint parameters were included in their models.)

To sum up, I have argued at length that regression approaches are superior to group mean and correlation coefficient comparisons for the purposes of testing the ‘flattened slope’ prediction. Acknowledging the reservations vis-à-vis self-estimated uas, we still find that while the relationship between aoa and ua is not necessarily perfectly linear in the studies discussed, the data do not lend unequivocal support to this prediction. In the following section, I will reanalyse data from a recent empirical paper on the cph by DeKeyser et al. [44]. The first goal of this reanalysis is to further illustrate some of the statistical fallacies encountered in cph studies. Second, by making the computer code available I hope to demonstrate how the relevant regression models, viz. piecewise regression models, can be fitted and how the aoa representing the optimal breakpoint can be identified. Lastly, the findings of this reanalysis will contribute to our understanding of how aoa affects ua as measured using a gjt.

## Materials

### Summary of DeKeyser et al. (2010)

I chose to reanalyse a recent empirical paper on the cph by DeKeyser et al. [44] (henceforth DK et al.). This paper lends itself well to a reanalysis since it exhibits two highly commendable qualities: the authors spell out their hypotheses lucidly and provide detailed numerical and graphical data descriptions. Moreover, the paper's lead author is very clear on what constitutes a necessary condition for accepting the cph: a non-linearity in the age of onset of acquisition (aoa)–ultimate attainment (ua) function, with ua declining less strongly as a function of aoa in older, post-cp arrivals compared to younger arrivals [14], [22]. Lastly, it claims to have found cross-linguistic evidence from two parallel studies backing the cph and should therefore be an unsuspected source to cph proponents.

DK et al. present data from comparable investigations into the relationship between aoa and ua in morphosyntactic judgements in two groups of adult Russian-speaking immigrants who had started learning English (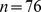) or Hebrew (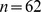) as an L2 at different ages in North America and Israel, respectively. The grammaticality judgement task (gjt) was adapted from Johnson and Newport's study [23] and was presented auditorily. For each of the 204 items, the participant had to indicate whether it was a permissible utterance in the respective L2 or not. One point was awarded for each correct answer. Participants were split up into three aoa groups: those who emigrated before the age of 18 (young), those between the ages of 18 and 40 (middle) and those who arrived after age 40 (old). In addition, all participants took a verbal aptitude test. For further details, I refer to the original publication.

The authors set out to test the following hypotheses:

- Hypothesis 1: For both the L2 English and the L2 Hebrew group, the slope of the age of arrival–ultimate attainment function will not be linear throughout the lifespan, but will instead show a marked flattening between adolescence and adulthood.
- Hypothesis 2: The relationship between aptitude and ultimate attainment will differ markedly for the young and older arrivals, with significance only for the latter. (DK et al., p. 417)

Both hypotheses were purportedly confirmed, which in the authors' view provides evidence in favour of cph. The problem with this conclusion, however, is that it is based on a comparison of correlation coefficients. As I have argued above, correlation coefficients are not to be confused with regression coefficients and cannot be used to directly address research hypotheses concerning slopes, such as Hypothesis 1. In what follows, I will reanalyse the relationship between DK et al.'s aoa and gjt data in order to address Hypothesis 1. Additionally, I will lay bare a problem with the way in which Hypothesis 2 was addressed. The extracted data and the computer code used for the reanalysis are provided as supplementary materials, allowing anyone interested to scrutinise and easily reproduce my whole analysis and carry out their own computations (see ‘supporting information’).

### Data extraction

DK et al. provided high-resolution scatterplots, downloadable from the journal's website, to illustrate the relationship between aoa and gjt performance. Using the open source program g3data, we extracted the data underlying these scatterplots. g3data is downloadable from https://github.com/pn2200/g3data and provides an interface in which users first identify the 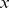
- and 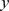-axes of a scatterplot and then point and click on the data points in it in order to extract the 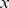
- and 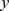-coordinates of the selected points. For the Israel study, we chose to round off the aoa data to the nearest integer, as was the case in the North America study, rather than to the first decimal, as in the original. The extracted North America and Israel data are supplied as Datasets S1 and S2, respectively.

In order to verify whether we did in fact extract the data points to a satisfactory degree of accuracy, I computed summary statistics for the extracted aoa and gjt data and checked these against the descriptive statistics provided by DK et al. (pp. 421 and 427). These summary statistics for the extracted data are presented in Table 1. In addition, I computed the correlation coefficients for the aoa–gjt relationship for the whole aoa range and for aoa-defined subgroups and checked these coefficients against those reported by DK et al. (pp. 423 and 428). The correlation coefficients computed using the extracted data are presented in Table 2. Both checks strongly suggest the extracted data to be virtually identical to the original data, and Dr DeKeyser confirmed this to be the case in response to an earlier draft of the present paper (personal communication, 6 May 2013).

**Table 1:** Descriptive statistics for the extracted data for the North America and Israel studies.

		Range	Mean	SD
North America	aoa	5–71	32.54	18.01
	gjt	104–198	150.76	27.32
Israel	aoa	4–65	30.55	16.95
	gjt	101–196	149.58	26.33

**Table 2:** Correlation coefficients for the relationship between AOA and GJT based on the extracted data for the North America and Israel studies.

	Overall	Young	Middle	Old
North America	−0.80 (76)	−069 (20)	−0.45 (26)	−0.27 (30)
Israel	−0.79 (62)	−0.46 (17)	−0.37 (32)	−0.54 (13)

Correlation coefficients are reported for the whole age range (‘Overall’) as well as for aoa-defined subgroups (as defined by DK et al.). Figures between brackets represent the number of participants in each cell.

## Results and Discussion

### Modelling the link between age of onset of acquisition and ultimate attainment

**Figure 3:**
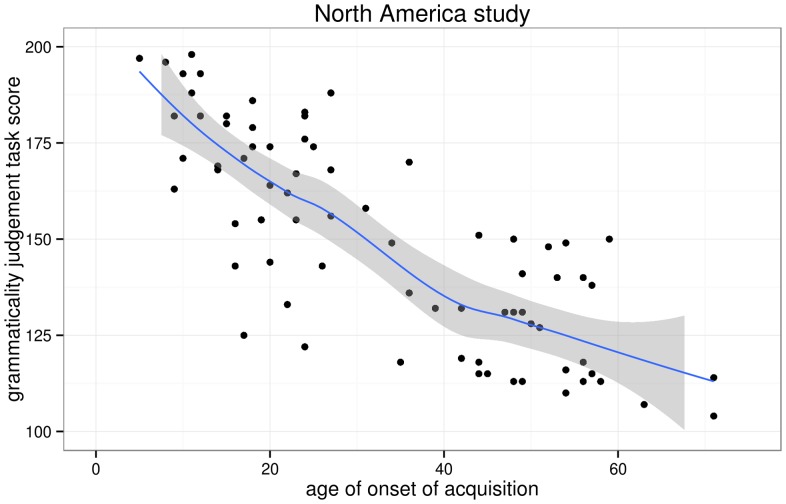
Scatterplot of the AOA–GJT relationship in the North America study. The trend line is a non-parametric scatterplot smoother. The scatterplot itself is a near-perfect replication of DK et al.'s Fig. 1.

**Figure 4:**
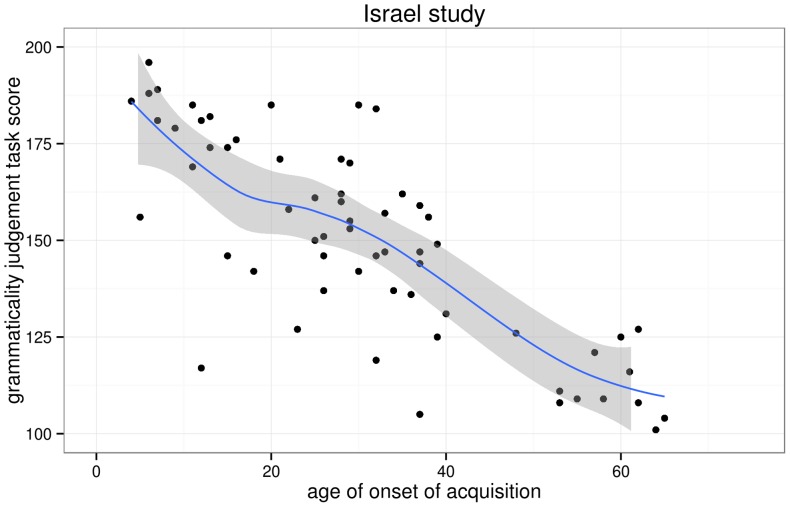
Scatterplot of the AOA–GJT relationship in the Israel study. The trend line is a non-parametric scatterplot smoother. The scatterplot itself is a near-perfect replication of DK et al.'s Fig. 5.

I first replotted the aoa and gjt data we extracted from DK et al.'s scatterplots and added non-parametric scatterplot smoothers in order to investigate whether any changes in slope in the aoa–gjt function could be revealed, as per Hypothesis 1. Figures 3 and 4 show this not to be the case. Indeed, simple linear regression models that model gjt as a function of aoa provide decent fits for both the North America and the Israel data, explaining 65% and 63% of the variance in gjt scores, respectively. The parameters of these models are given in Table 3.

**Table 3:** Linear regression models containing no breakpoints.

	Intercept ± SE	Slope ± SE	*R* ^2^	*F*-test of model fit
North America	168.50±2.42	−1.22±0.10	0.65	*F*(1.74) = 135.3, *P*<0.001
Israel	164.00±2.57	−1.23±0.12	0.63	*F*(1.60) = 100.4, *P*<0.001

gjt is modelled as a function of aoa. For ease of comparison with the breakpoint models, aoa was centred at 18 years.

These straightforward analyses do not reveal any support for Hypothesis 1. Still, for the sake of completeness, let us turn to the issue of determining whether slope differences between aoa groups, if they do indeed exist, are substantial enough to invoke a critical period. What needs to be established is whether including multiple slopes in a model contributes sufficiently to the fit of the model to the data to offset the loss of parsimony associated with a simpler one-slope model. To this end, I computed linear regression models allowing of breakpoints in the regression slope (‘piecewise regression models’). Similarly to ordinary regression, piecewise regression models the outcome variable 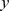 as a function of an overall intercept 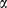 and a slope parameter 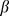 linking it to a predictor variable 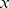. In contrast to ordinary regression, however, the 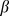 parameter of a piecewise regression model changes as a function of a binary indicator variable, which indicates whether 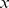 lies before or beyond the breakpoint to be modelled, 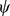:
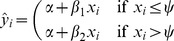
(4)


To ensure that both segments are joined at the breakpoint, the predictor variable is first centred at the breakpoint value, i.e. the breakpoint value is subtracted from the original predictor variable values. For a blow-by-blow account of how such models can be fitted in r, I refer to an example analysis by Baayen [55, pp. 214–222].

**Figure 5:**
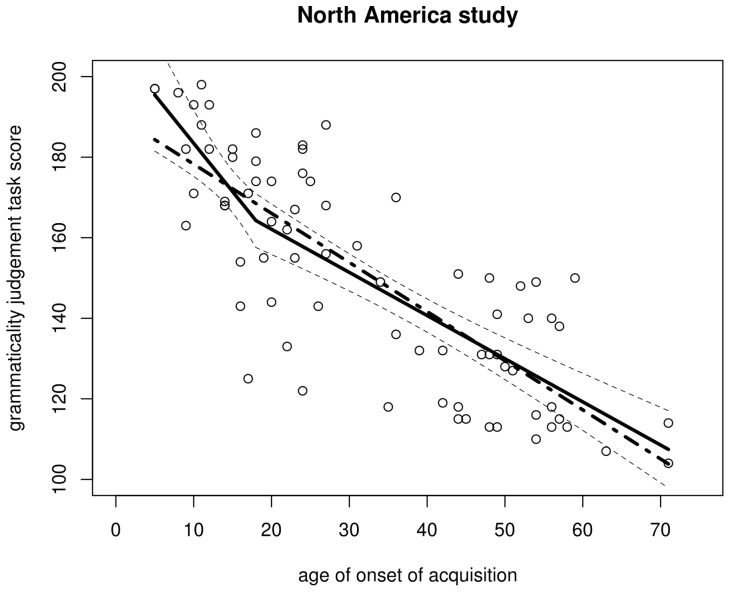
Regression lines for the North America data. Solid: regression with breakpoint at aoa 18 (dashed lines represent its 95% confidence interval); dot-dash: regression without breakpoint.

**Figure 6:**
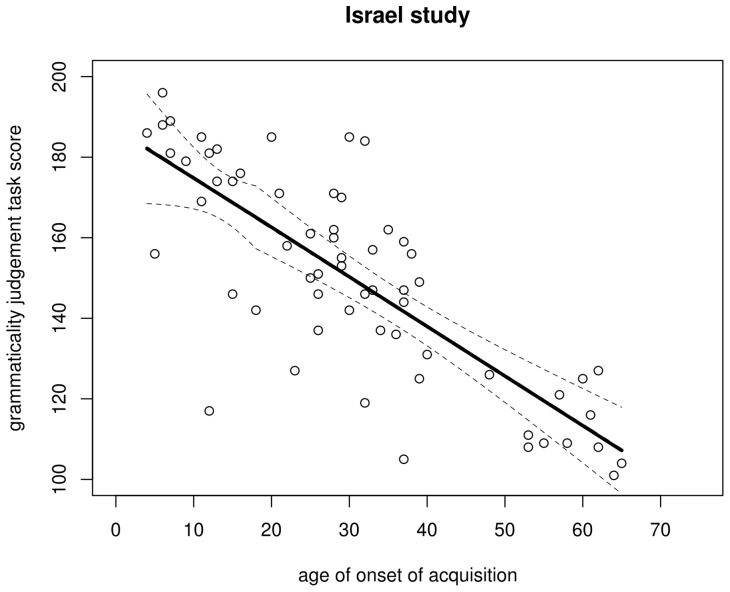
Regression lines for the Israel data. Solid: regression with breakpoint at aoa 18 (dashed lines represent its 95% confidence interval); dot-dash (hardly visible due to near-complete overlap): regression without breakpoint.

For the first models, I set the breakpoint at aoa 18, the cut-off used by DK et al. The models' details are presented in Table 4. At first glance, the coefficients for the North America study in Table 4 might appear to confirm Hypothesis 1: the slope linking aoa and gjt is flatter for participants with aoa 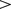
18 than for those with aoa 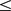
18. However, as Figure 5 illustrates, the regression line of a model without a breakpoint falls well within the 95% confidence interval of the regression line of the breakpoint model. Thus, the breakpoint parameter may be superfluous. For the Israel data, the change in slope at the breakpoint is hardly perceptible and the regression lines plotted in Figure 6 overlap almost completely. In both cases, the inclusion of a breakpoint parameter at aoa 18 leads to an at most negligible increase in variance accounted for (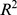). Unsurprisingly, then, formal 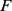-tests confirm that the simpler models, i.e. the ones that do not include breakpoints at aoa 18, are to be preferred on the grounds of parsimony (North America: 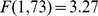, 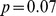; Israel: 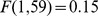, 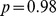). Note, incidentally, that 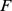-tests compare whether the residual sums of squares associated with the more complex model is smaller than the residual sums of squares associated with the simpler model. As such, they are one-tailed tests. Halving the 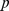-value of the North America model comparison to take into account that DK et al. predicted the direction of the change in slope at the breakpoint and thereby achieving significance at 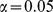 is therefore unsound.

**Table 4:** Linear regression models containing breakpoints at AOA 18.

	Intercept ± SE	Slope ± SE (aoa ≤18)	Slope ± SE (aoa >18)	*R* ^2^	*F*-test of model fit
North America	164.24±3.35	−2.40±0.66	−1.07±0.13	0.66	*F*(2.73) = 71.4, *P*<0.001
Israel	165.07±3.90	−1.21±0.62	−1.23±0.17	0.63	*F*(2.59) = 49.4, *P*<0.001

gjt is modelled as a function of aoa. Following Baayen [55, pp. 214–222], aoa was centred at 18 years.

**Figure 7:**
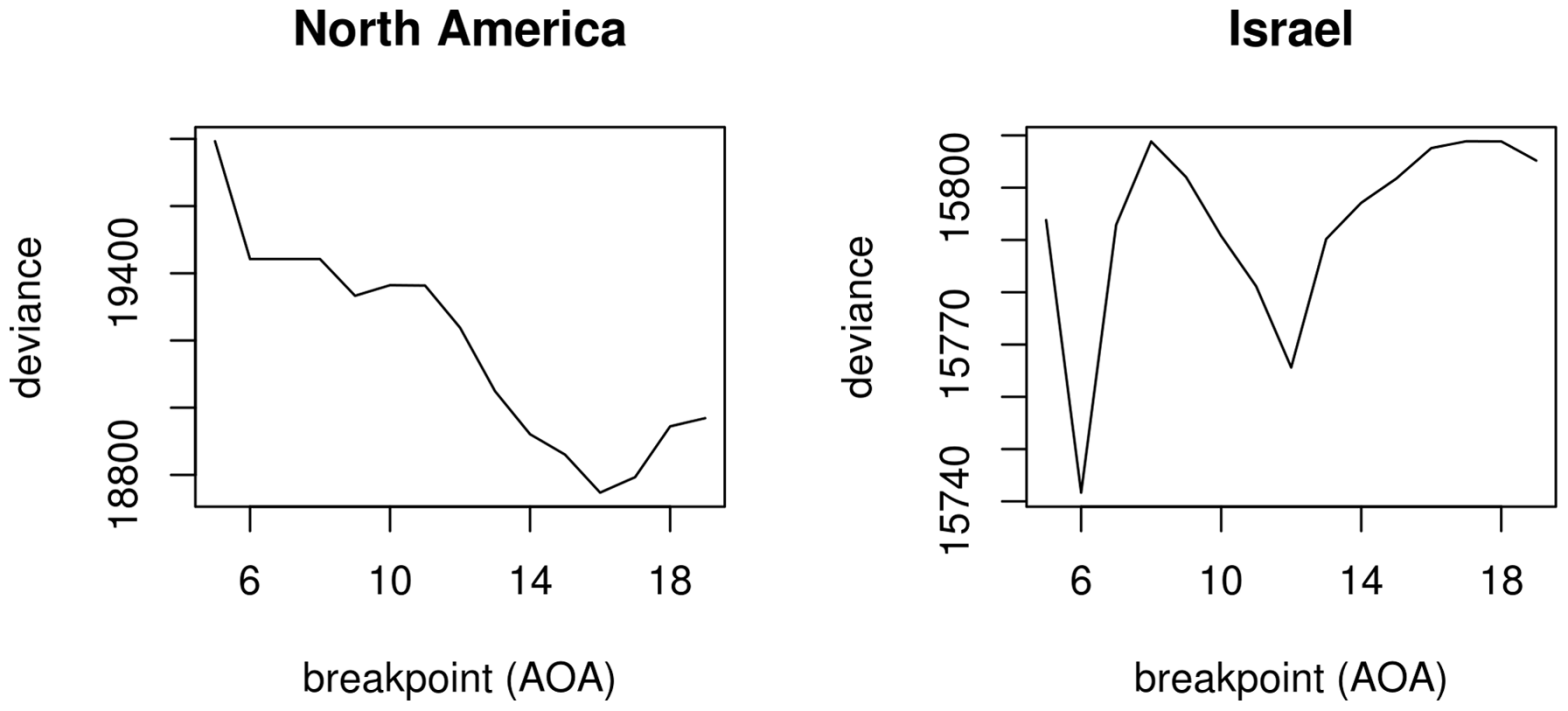
Deviances for regression models assuming breakpoints as a function of the position of the breakpoints.

**Figure 8:**
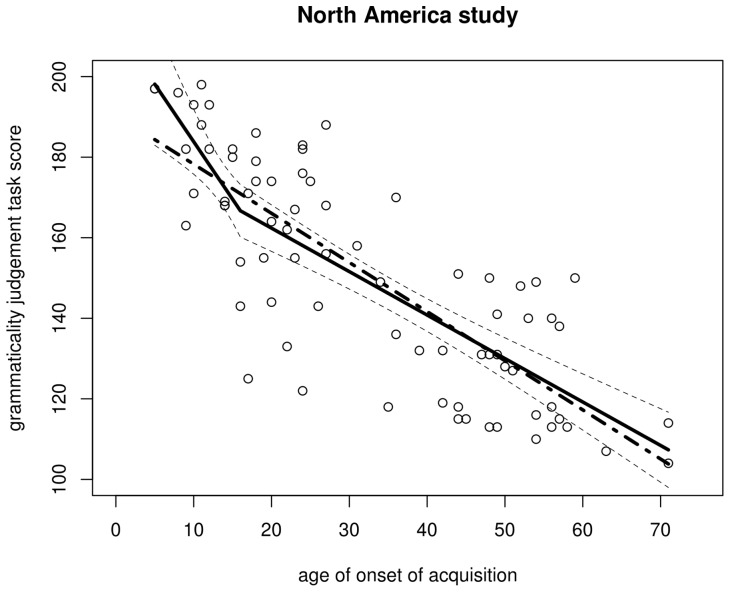
Regression lines for the North America data. Solid: regression with breakpoint at aoa 16 (dashed lines represent its 95% confidence interval); dot-dash: regression without breakpoint.

**Figure 9:**
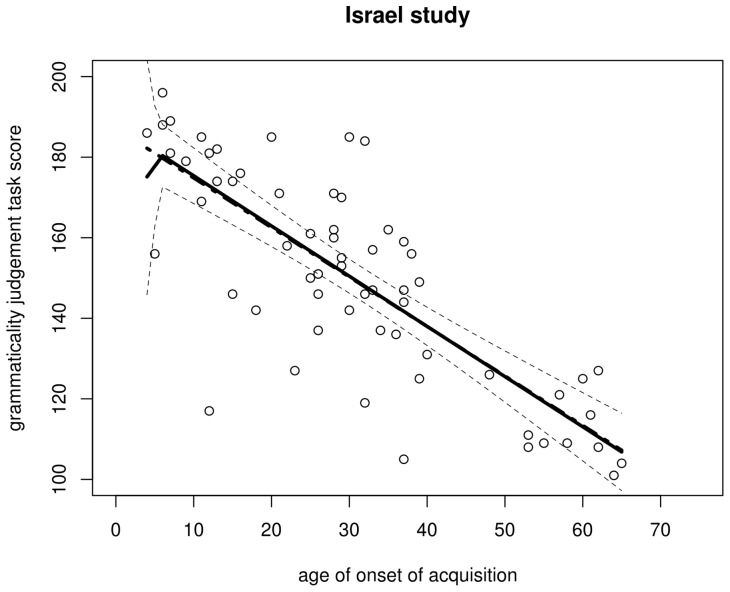
Regression lines for the Israel data. Solid: regression with breakpoint at aoa 6 (dashed lines represent its 95% confidence interval); dot-dash (hardly visible due to near-complete overlap): regression without breakpoint.

Having ascertained that the inclusion of a breakpoint in the regression to mark the end of a putative critical period at aoa 18 years does not improve the fit of the model to the data, we are still left with the possibility that placing the breakpoint *elsewhere* might do so (see the discussion on the cph's scope above). Following Baayen [55], I computed a series of regression models for both data sets in which the position of the breakpoints varied between aoa 5 and 19 years. Breakpoints at aoa 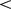
5 could not be fitted for lack of data points and breakpoints at aoa 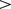
19 would hardly be indicative of a critical period that ends before maturity. For each fitted model, the deviance (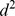) was computed, i.e. the sum of the squared differences between the actual data points and the values predicted by the model: 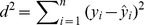. The smaller the deviance, the better the model fits the data. Thus, the optimal breakpoint is the one that results in the model with the smallest deviance. The fitted breakpoints and their associated deviances are plotted in Figure 7. For the North America study, a breakpoint at aoa 16 years is optimal; for the Israel study, the optimal breakpoint lies at aoa 6 years. The regression models were then refitted with and without breakpoints at aoa 16 (North America) and 6 (Israel). The models' details are presented in Tables 5 and 6 for the North America study and Tables 7 and 8 for the Israel study. The conclusions are largely, though not completely identical compared to when the breakpoints were fixed at aoa 18. For the North America study, the slope flattens after the breakpoint, but as Figure 8 shows, the regression line for a model without a breakpoint still falls entirely within the 95% confidence interval of the breakpoint model and the increase in 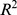 is small. Nonetheless, an 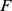-test returns a borderline significant 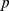-value, which can be taken as support for the breakpoint model (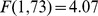, 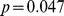) Two other relative goodness-of-fit measures likewise produce borderline results: the breakpoint model has a slightly better (i.e. lower) Akaike Information Criterion [53] value than the simpler model (642.3 and 644.4, respectively), but a slightly worse (i.e. higher) Bayesian Information Criterion [54] value (651.6 and 651.4, respectively). For the Israel study, both regression lines again show almost complete overlap (Figure 9) and the increase in 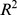 is negligible. Unsurprisingly, an 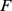-test yields no support for the breakpoint model (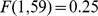, 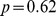).

**Table 5:** Regression model for the North America data without a breakpoint at AOA 16.

Intercept ± SE	Slope ± SE	*R* ^2^	*F*-test of model fit
170.94±2.56	−1.22±0.10	0.65	*F*(1,74) = 135.3, *P*<0.001

gjt is modelled as a function of aoa. aoa was centred at 16 years.

**Table 6:** Regression model for the North America data with a breakpoint at AOA 16.

Intercept ± SE	Slope ± SE (aoa ≤16)	Slope ± SE (aoa >16)	*R* ^2^	*F*-test of model fit
166.69±3.27	−2.86±082	−1.08±0.12	0.67	*F*(2,73 = 72.5), *P*<0.001

gjt is modelled as a function of aoa. aoa was centred at 16 years.

**Table 7:** Regression model for the Israel data without a breakpoint at AOA 6.

Intercept ± SE	Slope ± SE	*R* ^2^	*F*-test of model fit
179.75±3.65	−1.23±0.12	0.63	*F*(1,60) = 100.4, *P*<0 001

gjt is modelled as a function of aoa. aoa was centred at 6 years.

**Table 8:** Regression model for the Israel data with a breakpoint at AOA 6.

Intercept ± SE	slope ± SE (aoa <6)	Slope ± SE (aoa >6)	*R* ^2^	*F*-test of model fit
180.37±3.87	2.62±7.67	−1.25±0.13	0.63	*F*(2,59) = 49.7, *p*<0.001

gjt is modelled as a function of aoa. aoa was centred at 6 years.

As a technical sidebar, note that regression models are ideally fitted on homoscedastic data, meaning that the variance around the model's predictions does not vary as a function of the value of those predictions. This condition is not fully met in the present data sets and, indeed, heteroscedasticity seems to be endemic in research on age effects: ua variance is typically larger in the older age groups. A first option to deal with heteroscedasticity of this kind is to fit robust regression models (see [56]) both with and without breakpoints using the rlm() function in the MASS package for R [57]. The parameters of these models were highly similar to those of their ordinary counterparts (see Script S1). An alternative is to specify the distribution of the residuals in a generalised least squares model (see Chapter 4 in [58] for an accessible applied introduction). In the ordinary linear model, the residuals are assumed to come from a normal distribution with mean 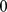 and fixed variance 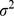: 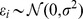. In generalised least squares models, the variance part can be specified to be dependent on a covariate or on the fitted values. In the present case, the variance of the normal distribution from which the residuals are drawn can be specified to increase linearly with the aoa covariate: 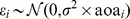. Generalised least squares models with this variance structure were fitted both with and without breakpoints using the gls() function in the nlme package for R [59], but doing so did not alter the conclusions either (see Script S1).

In sum, a regression model that allows for changes in the slope of the the aoa–gjt function to account for putative critical period effects provides a somewhat better fit to the North American data than does an everyday simple regression model. The improvement in model fit is marginal, however, and including a breakpoint does not result in any detectable improvement of model fit to the Israel data whatsoever. Breakpoint models therefore fail to provide solid cross-linguistic support in favour of critical period effects: across both data sets, gjt can satisfactorily be modelled as a linear function of aoa.

### On partialling out ‘age at testing’

As I have argued above, correlation coefficients cannot be used to test hypotheses about slopes. When the correct procedure is carried out on DK et al.'s data, no cross-linguistically robust evidence for changes in the aoa–gjt function was found. In addition to comparing the zero-order correlations between aoa and gjt, however, DK et al. computed partial correlations in which the variance in aoa associated with the participants' age at testing (aat; a potentially confounding variable) was filtered out. They found that these partial correlations between aoa and gjt, which are given in Table 9, differed between age groups in that they are stronger for younger than for older participants. This, DK et al. argue, constitutes additional evidence in favour of the cph. At this point, I can no longer provide my own analysis of DK et al.'s data seeing as the pertinent data points were not plotted. Nevertheless, the detailed descriptions by DK et al. strongly suggest that the use of these partial correlations is highly problematic. Most importantly, and to reiterate, correlations (whether zero-order or partial ones) are actually of no use when testing hypotheses concerning slopes. Still, one may wonder why the partial correlations differ across age groups. My surmise is that these differences are at least partly the by-product of an imbalance in the sampling procedure.

**Table 9:** Partial correlation coefficients for the relationship between AOA and GJT with AAT controlled for.

	Overall	Young	Middle	Old
North America	−0.29 (76)	−0.71 (20)	−0.17 (26)	−0.12 (30)
Israel	−0.28 (62)	−0.51 (17)	−0.12 (32)	−0.33 (13)

Coefficients for separate age groups as reported by DK et al., pp. 423 and 429; overall coefficients were computed on the basis of the data in DK et al., Tables 2 and 4. Figures between brackets represent the number of participants in each cell.

As indicated in DK et al.'s Tables 2 and 4 (pp. 424 and 430), aoa and aat are correlated to the point of near-unity (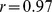 and 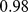). aat and gjt, too, are highly correlated (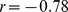 and 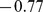) and the aat–gjt correlations are nearly identical in magnitude to the aoa–gjt correlations (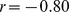 and 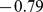). In other words, aoa and aat essentially represent the same variable when the whole aoa range is considered. However, DK et al. did not compute their partial correlations on the basis of the whole aoa continuum. Rather, they looked at each aoa slice separately. Crucially, however, the aoa–aat correlation was not constant across aoa groups. In the North America study, for instance, aoa and aat correlated less strongly in the youngest arrivals (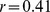) than in the older arrivals (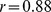 and 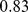); in the Israel study, the correlations were rather more comparable (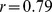, 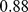 and 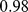). What DK et al. did not take into account is that it is these differences in the strength of the aoa–aat correlation, which for the purposes of testing the cph are completely uninteresting, that are largely responsible for the differences in the strength of the partial correlations: partial correlations decrease as the correlations between the ‘independent’ variables increase [60]. The partial correlation between 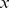 and 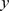 controlling for 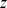 (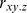) is computed solely on the basis of the underlying zero-order correlations 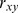, 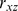 and 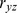:
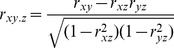
(5)


Decreasing partial correlations with increasing zero-order correlations between the independent variables (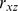) follow naturally from this function. This is the most straightforward explanation of why the differences in the partial correlations are smaller between all the groups in the Israel study compared to the North America study: the aoa–aat correlations in the Israel study are high in all age groups, not just in two of them.

The upshot of this brief discussion is that the partial correlation differences reported by DK et al. are at least partly the result of an imbalance in the sampling procedure: aoa and aat were simply less intimately tied for the young arrivals in the North America study than for the older arrivals with L2 English or for all of the L2 Hebrew participants. In an ideal world, we would like to fix aat or ascertain that it at most only weakly correlates with aoa. This, however, would result in a strong correlation between aoa and another potential confound variable, length of residence in the L2 environment, bringing us back to square one. Allowing for only moderate correlations between aoa and aat might improve our predicament somewhat, but even in that case, we should tread lightly when making inferences on the basis of statistical control procedures [61].

### On estimating the role of aptitude

Having shown that Hypothesis 1 could not be confirmed, I now turn to Hypothesis 2, which predicts a differential role of aptitude for ua in sla in different aoa groups. More specifically, it states that the correlation between aptitude and gjt performance will be significant only for older arrivals. The correlation coefficients of the relationship between aptitude and gjt are presented in Table 10.

**Table 10:** Correlation coefficients for the relationship between aptitude and GJT.

	Overall	Young	Middle	Old
North America	0.210 (76)	0.11 (20)	0.44 (26)	0.33 (30)
Israel	0.00 (62)	−0.37 (17)	0.45 (32)	0.14 (13)

Data are as reported by DK et al., pp. 425 and 429. Figures between brackets represent the number of participants in each cell.

The problem with both the wording of Hypothesis 2 and the way in which it is addressed is the following: it is assumed that a variable has a reliably different effect in different groups when the effect reaches significance in one group but not in the other. This logic is fairly widespread within several scientific disciplines (see e.g. [62] for a discussion). Nonetheless, it is demonstrably fallacious [63]. Here we will illustrate the fallacy for the specific case of comparing two correlation coefficients.

The 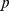-value associated with a correlation coefficient is solely a function of three factors: (a) the strength of the correlation, i.e. 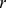, (b) the number of pairs of correlated observations, i.e. 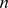, and (c) whether one wishes to conduct a one-tailed or a two-tailed test. Given 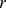 and 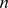, a 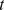-statistic can be calculated using the formula 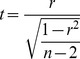. A two-tailed 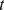-test reaches significance at 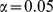 at around 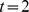, though the precise figure varies as a function of 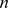. Thus, a two-tailed test will reveal that a correlation coefficient of 0.28 for 50 pairs of observations is significant at the 0.05 level (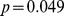), whereas a coefficient of 0.27 fails to reach significance (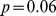). But it would be clearly wrong to conclude that the minuscule difference between the two correlation coefficients therefore has to be significant, too. Most researchers will probably share this insight when 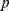-values hover around the 0.05 threshold. Importantly, however, even more substantially different correlation coefficients are not necessarily ‘significantly different’ from one another either, not even if their associated 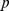-values indicated that one were ‘highly significant’ (e.g. 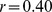, 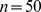, 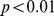) and the other were not even close to significance (e.g. 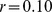, 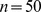, 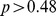).

The correct procedure for comparing independent correlation coefficients consists of converting the correlation coefficients to 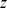-scores using Fisher's 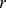-to-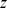 transformation (Eq. 6), computing the 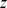-statistic of the difference between the two converted correlation coefficients (Eq. 7) and checking this 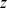-statistic for significance [64], [65]. In two-tailed tests, the difference of two correlation coefficients reaches significance at 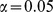 for 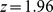; in one-tailed tests for 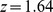.
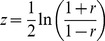
(6)

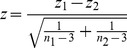
(7)


Plugging in the numbers for the substantially different correlation coefficients of 0.10 and 0.40 introduced above, we find that the difference between these coefficients is actually not significant (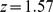). When the correct method is applied to DK et al.'s correlation coefficients, comparisons of the young arrivals against the older arrivals only revealed a single difference in correlation strength, even when carrying out one-tailed tests: the correlation between aptitude and gjt in young arrivals in Israel (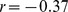, 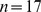) differs significantly from the one in middle-aged arrivals (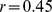, 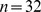; 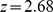, one-tailed 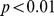). For all other comparisons, 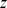 varied between 0.46 and 1.28.

Apart from not being replicated in the North America study, does this difference actually show anything? I contend that it does not: what is of interest are not so much the correlation coefficients, but rather the interactions between aoa and aptitude in models predicting gjt. These interactions could be investigated by fitting a multiple regression model in which the postulated cp breakpoint governs the slope of both aoa and aptitude. If such a model provided a substantially better fit to the data than a model without a breakpoint for the aptitude slope *and* if the aptitude slope changes in the expected direction (i.e. a steeper slope for post-cp than for younger arrivals) for different L1–L2 pairings, only then would this particular prediction of the cph be borne out.

## Discussion

Using data extracted from a paper reporting on two recent studies that purport to provide evidence in favour of the cph and that, according to its authors, represent a major improvement over earlier studies (DK et al., p. 417), it was found that neither of its two hypotheses were actually confirmed when using the proper statistical tools. As a matter of fact, the gjt scores continue to decline at essentially the same rate even beyond the end of the putative critical period. According to the paper's lead author, such a finding represents a serious problem to his conceptualisation of the cph [14]). Moreover, although modelling a breakpoint representing the end of a cp at aoa 16 may improve the statistical model slightly in study on learners of English in North America, the study on learners of Hebrew in Israel fails to confirm this finding. In fact, even if we were to accept the optimal breakpoint computed for the Israel study, it lies at aoa 6 and is associated with a different geometrical pattern.

Diverging age trends in parallel studies with participants with different L2s have similarly been reported by Birdsong and Molis [26] and are at odds with an L2-independent cph. One parsimonious explanation of such conflicting age trends may be that the overall, cross-linguistic age trend is in fact linear, but that fluctuations in the data (due to factors unaccounted for or randomness) may sometimes give rise to a ‘stretched L’-shaped pattern (Figure 1, left panel) and sometimes to a ‘stretched 7’-shaped pattern (Figure 1, middle panel; see also [66] for a similar comment).

Importantly, the criticism that DeKeyser and Larsson-Hall levy against two studies reporting findings similar to the present [48], [49], viz. that the data consisted of self-ratings of questionable validity [14], does not apply to the present data set. In addition, DK et al. did not exclude any outliers from their analyses, so I assume that DeKeyser and Larsson-Hall's criticism [14] of Birdsong and Molis's study [26], i.e. that the findings were due to the influence of outliers, is not applicable to the present data either. For good measure, however, I refitted the regression models with and without breakpoints after excluding one potentially problematic data point per model. The following data points had absolute standardised residuals larger than 2.5 in the original models without breakpoints as well as in those with breakpoints: the participant with aoa 17 and a gjt score of 125 in the North America study and the participant with aoa 12 and a gjt score of 117 in the Israel study. The resultant models were virtually identical to the original models (see Script S1). Furthermore, the aoa variable was sufficiently fine-grained and the aoa–gjt curve was not ‘presmoothed’ by the prior aggregation of gjt across parts of the aoa range (see [51] for such a criticism of another study). Lastly, seven of the nine “problems with supposed counter-evidence” to the cph discussed by Long [5] do not apply either, viz. (1) “[c]onfusion of rate and ultimate attainment”, (2) “[i]nappropriate choice of subjects”, (3) “[m]easurement of AO”, (4) “[l]eading instructions to raters”, (6) “[u]se of markedly non-native samples making near-native samples more likely to sound native to raters”, (7) “[u]nreliable or invalid measures”, and (8) “[i]nappropriate L1–L2 pairings”. Problem No. 5 (“Assessments based on limited samples and/or “language-like” behavior”) may be apropos given that only gjt data were used, leaving open the theoretical possibility that other measures might have yielded a different outcome. Finally, problem No. 9 (“Faulty interpretation of statistical patterns”) is, of course, precisely what I have turned the spotlights on.

## Conclusions

The critical period hypothesis remains a hotly contested issue in the psycholinguistics of second-language acquisition. Discussions about the impact of empirical findings on the tenability of the cph generally revolve around the reliability of the data gathered (e.g. [5], [14], [22], [52], [67], [68]) and such methodological critiques are of course highly desirable. Furthermore, the debate often centres on the question of exactly what version of the cph is being vindicated or debunked. These versions differ mainly in terms of its scope, specifically with regard to the relevant age span, setting and language area, and the testable predictions they make. But even when the cph's scope is clearly demarcated and its main prediction is spelt out lucidly, the issue remains to what extent the empirical findings can actually be marshalled in support of the relevant cph version. As I have shown in this paper, empirical data have often been taken to support cph versions predicting that the relationship between age of acquisition and ultimate attainment is not strictly linear, even though the statistical tools most commonly used (notably group mean and correlation coefficient comparisons) were, crudely put, irrelevant to this prediction. Methods that are arguably valid, e.g. piecewise regression and scatterplot smoothing, have been used in some studies [21], [26], [49], but these studies have been criticised on other grounds. To my knowledge, such methods have never been used by scholars who explicitly subscribe to the cph.

I suspect that what may be going on is a form of ‘confirmation bias’ [69], a cognitive bias at play in diverse branches of human knowledge seeking: Findings judged to be consistent with one's own hypothesis are hardly questioned, whereas findings inconsistent with one's own hypothesis are scrutinised much more strongly and criticised on all sorts of points [70]–[73]. My reanalysis of DK et al.'s recent paper may be a case in point. cph exponents used correlation coefficients to address their prediction about the slope of a function, as had been done in a host of earlier studies. Finding a result that squared with their expectations, they did not question the technical validity of their results, or at least they did not report this. (In fact, my reanalysis is actually a case in point in two respects: for an earlier draft of this paper, I had computed the optimal position of the breakpoints incorrectly, resulting in an insignificant improvement of model fit for the North American data rather than a borderline significant one. Finding a result that squared with my expectations, *I* did not question the technical validity of *my* results – until this error was kindly pointed out to me by Martijn Wieling (University of Tübingen).) That said, I am keen to point out that the statistical analyses in this particular paper, though suboptimal, are, as far as I could gather, reported correctly, i.e. the confirmation bias does not seem to have resulted in the blatant misreportings found elsewhere (see [74] for empirical evidence and discussion). An additional point to these authors' credit is that, apart from explicitly identifying their cph version's scope and making crystal-clear predictions, they present data descriptions that actually permit quantitative reassessments and have a history of doing so (e.g. the appendix in [8]). This leads me to believe that they analysed their data all in good conscience and to hope that they, too, will conclude that their own data do not, in fact, support their hypothesis.

I end this paper on an upbeat note. Even though I have argued that the analytical tools employed in cph research generally leave much to be desired, the original data are, so I hope, still available. This provides researchers, cph supporters and sceptics alike, with an exciting opportunity to reanalyse their data sets using the tools outlined in the present paper and publish their findings at minimal cost of time and resources (for instance, as a comment to this paper). I would therefore encourage scholars to engage their old data sets and to communicate their analyses openly, e.g. by voluntarily publishing their data and computer code alongside their articles or comments. Ideally, cph supporters and sceptics would join forces to agree on a protocol for a high-powered study in order to provide a truly convincing answer to a core issue in sla.

## Supporting Information

Dataset S1 **aoa and gjt data extracted from DeKeyser et al.'s North America study.**
(CSV)

Dataset S2 **aoa and gjt data extracted from DeKeyser et al.'s Israel study.**
(CSV)

Script S1 **Script with annotated R code used for the reanalysis.** All add-on packages used can be installed from within R.
(R) Click here for additional data file.

## References

[1] Penfield W, Roberts L (1959) Speech and brain mechanisms. Princeton: Princeton University Press.

[2] Lenneberg EH (1967) Biological foundations of language. New York: Wiley.

[3] Singleton D (2007) The critical period hypothesis: Some problems. Interlingüística 17: 48–56.

[4] Singleton D (2005) The critical period hypothesis: A coat of many colours. International Review of Applied Linguistics in Language Teaching 43: 269–285.

[5] Long MH (2005) Problems with supposed counter-evidence to the critical period hypothesis. International Review of Applied Linguistics in Language Teaching 43: 287–317.

[6] Muñoz C, Singleton D (2011) A critical review of age-related research on L2 ultimate attainment. Language Teaching 44: 1–35.

[7] Ruben RJ (1997) A time frame of critical/sensitive periods of language development. Acta Otolaryngologica 117: 202–205. 10.3109/00016489709117769 9105448

[8] DeKeyser R (2000) The robustness of critical period effects in second language acquisition. Studies in Second Language Acquisition 22: 499–533.

[9] Birdsong D (2006) Age and second language acquisition and processing: A selective overview. Language Learning 56: 9–49.

[10] Long MH (2007) Problems in SLA. Mahwah, NJ: Lawrence Erlbaum.

[11] Krashen SD, Long MH, Scarcella RC (1979) Age, rate and eventual attainment in second language acquisition. TESOL Quarterly 13: 573–582.

[12] Snow CE, Hoefnagel-Höhle M (1977) Age differences in the pronunciation of foreign sounds. Language and Speech 20: 357–365. 616512 10.1177/002383097702000407

[13] Snow CE, Hoefnagel-Höhle M (1978) The critical period for language acquisition: Evidence from second language learning. Child Development 49: 1114–1128.

[14] DeKeyser R, Larson-Hall J (2005) What does the critical period really mean? In: Kroll and De Groot [75], 88–108.

[15] Abrahamsson N, Hyltenstam K (2009) Age of onset and nativelikeness in a second language: Listener perception versus linguistic scrutiny. Language Learning 59: 249–306.

[16] White L, Genesee F (1996) How native is near-native? The issue of ultimate attainment in adult second language acquisition. Second Language Research 12: 233–265.

[17] Cook VJ (1992) Evidence for multicompetence. Language Learning 42: 557–591.

[18] Grosjean F (1989) Neurolinguists, beware! The bilingual is not two monolinguals in one person. Brain and Language 36: 3–15. 2465057 10.1016/0093-934x(89)90048-5

[19] Newport EL (1991) Contrasting conceptions of the critical period for language. In: Carey S, Gelman R, editors, The epigenesis of mind: Essays on biology and cognition, Hillsdale, NJ: Lawrence Erlbaum. 111–130.

[20] Birdsong D (2005) Interpreting age effects in second language acquisition. In: Kroll and De Groot [75], 109–127.

[21] Hakuta K, Bialystok E, Wiley E (2003) Critical evidence: A test of the critical-period hypothesis for second-language acquisition. Psychological Science 14: 31–38. 12564751 10.1111/1467-9280.01415

[22] DeKeyser R (2012) Age effects in second language learning. In: Gass SM, Mackey A, editors, The Routledge handbook of second language acquisition, London: Routledge. 442–460.

[23] Johnson JS, Newport EL (1989) Critical period effects in second language learning: The inuence of maturational state on the acquisition of English as a second language. Cognitive Psychology 21: 60–99. 2920538 10.1016/0010-0285(89)90003-0

[24] Weisstein EW. Discontinuity. From *MathWorld*–A Wolfram Web Resource. Available: http://mathworld.wolfram.com/Discontinuity.html. Accessed 2012 March 2.

[25] Bialystok E, Miller B (1999) The problem of age in second-language acquisition: Inuences from language, structure, and task. Bilingualism: Language and Cognition 2: 127–145.

[26] Birdsong D, Molis M (2001) On the evidence for maturational constraints in second-language acquisition. Journal of Memory and Language 44: 235–249.

[27] Flege JE (1999) Age of learning and second language speech. In: Birdsong [76], 101–132.

[28] Flege JE, Yeni-Komshian GH, Liu S (1999) Age constraints on second-language acquisition. Journal of Memory and Language 41: 78–104.

[29] Johnson JS (1992) Critical period effects in second language acquisition: The effect of written versus auditory materials on the assessment of grammatical competence. Language Learning 42: 217–248.

[30] McDonald JL (2000) Grammaticality judgments in a second language: Inuences of age of acquisition and native language. Applied Psycholinguistics 21: 395–423.

[31] Patkowski MS (1980) The sensitive period for the acquisition of syntax in a second language. Language Learning 30: 449–472.

[32] Cohen J (1983) The cost of dichotomization. Applied Psychological Measurement 7: 249–253.

[33] Schmidt FL (1996) Statistical significance testing and cumulative knowledge in psychology: Implications for training of researchers. Psychological Methods 1: 115–129.

[34] Sedlmeier P, Gigerenzer G (1989) Do studies of statistical power have an effect on the power of studies? Psychological Bulletin 105: 309–316.

[35] Cohen J (1992) A power primer. Psychological Bulletin 112: 155–159. 19565683 10.1037//0033-2909.112.1.155

[36] Champely S (2009) pwr: Basic functions for power analysis. Available: http://cran.r-project.org/package=pwr. R package, version 1.1.1.

[37] R Core Team (2013) R: A language and environment for statistical computing. Available: http://www.r-project.org/. Software, version 2.15.3.

[38] Nakawaga S (2004) A farewell to Bonferroni: the problems of low statistical power and publication bias. Behavioral Ecology 15: 1044–1045.

[39] Perneger TV (1998) What's wrong with Bonferroni adjustments. BMJ 316: 1236–1238. 9553006 10.1136/bmj.316.7139.1236 PMC1112991

[40] Cohen J (1994) The Earth is round (p<05). American Psychologist 49: 997–1003.

[41] Ioannidis JPA (2005) Why most published research findings are false. PLoS Medicine 2: e124. 16060722 10.1371/journal.pmed.0020124 PMC1182327

[42] Simmons JP, Nelson LD, Simonsohn U (2011) False-positive psychology: Undisclosed exibility in data collection and analysis allows presenting anything as significant. Psychological Science 22: 1359–1366. 22006061 10.1177/0956797611417632

[43] Wetzels R, Matzke D, Lee MD, Rounder JN, Iverson GJ, et al (2011) Statistical evidence in experimental psychology: An empirical comparison using 855 t tests. Perspectives on Psychological Science 6: 291–298. 26168519 10.1177/1745691611406923

[44] DeKeyser R, Alfi-Shabtay I, Ravid D (2010) Cross-linguistic evidence for the nature of age effects in second language acquisition. Applied Psycholinguistics 31: 413–438.

[45] Flege JE, Birdsong D, Bialystok E, Mack M, Sung H, et al (2006) Degree of foreign accent in English sentences produced by Korean children and adults. Journal of Phonetics 34: 153–175.

[46] Marinova-Todd SH, Marshall DB, Snow CE (2000) Three misconceptions about age and L2 learning. TESOL Quarterly 34: 9–34.

[47] Hyltenstam K, Abrahamsson N (2003) Maturational constraints in sla. In: Doughty CJ, Long MH, editors, The handbook of second language acquisition, Malden, MA: Blackwell. 539–588.

[48] Stevens G (1999) Age of immigration and second language proficiency among foreign-born adults. Language in Society 28: 555–578.

[49] Bialystok E, Hakuta K (1999) Confounded age: Linguistic and cognitive factors in age differences for second language acquisition. In: Birdsong [76], 161–181.

[50] Cleveland WS (1979) Robust locally weighted regression and smoothing scatterplots. Journal of the American Statistical Association 74: 829–836.

[51] Stevens G (2004) Using census data to test the critical-period hypothesis for second-language acquisition. Psychological Science 15: 215–216. 15016295 10.1111/j.0956-7976.2004.01503012.x

[52] DeKeyser R (2006) A critique of recent arguments against the critical period hypothesis. In: Abello-Contesse C, Chacón-Beltrán R, López-Jiménez MD, Torreblanca-López MM, editors, Age in L2 acquisition and teaching, Bern: Peter Lang. 49–58.

[53] Akaike H (1974) A new look at the statistical model identification. IEEE Transactions on Automatic Control AC-19: 716–723.

[54] Schwarz G (1978) Estimating the dimension of a model. The Annals of Statistics 6: 461–464.

[55] Baayen RH (2008) Analyzing linguistic data: A practical introduction to statistics using R. Cambridge: Cambridge University Press.

[56] Fox J (2002) Robust regression. Appendix to An R and S-Plus Companion to Applied Regression. Available: http://cran.r-project.org/doc/contrib/Fox-Companion/appendix.html.

[57] Ripley B, Hornik K, Gebhardt A, Firth D (2012) MASS: Support functions and datasets for Venables and Ripley's MASS. Available: http://cran.r-project.org/package=MASS. R package, version 7.3–17.

[58] Zuur AF, Ieno EN, Walker NJ, Saveliev AA, Smith GM (2009) Mixed effects models and extensions in ecology with R. New York: Springer.

[59] Pinheiro J, Bates D, DebRoy S, Sarkar D, R Core Team (2013) nlme: Linear and nonlinear mixed effects models. Available: http://cran.r-project.org/package=nlme. R package, version 3.1–108.

[60] Blalock HM Jr (1963) Correlated independent variables: The problem of multicollinearity. Social Forces 42: 233–237.

[61] Christenfeld NJS, Sloan RP, Carroll D, Greenland S (2004) Risk factors, confounding, and the illusion of statistical control. Psychosomatic Medicine 66: 868–875. 15564351 10.1097/01.psy.0000140008.70959.41

[62] Nieuwenhuis S, Forstmann BU, Wagenmakers EJ (2011) Erroneous analyses of interactions in neuroscience: A problem of significance. Nature Neuroscience 14: 1105–1107. 21878926 10.1038/nn.2886

[63] Gelman A, Stern H (2006) The difference between “significant” and “not significant” is not itself statistically significant. The American Statistician 60: 328–331.

[64] Olkin I, Finn JD (1995) Correlations redux. Psychological Bulletin 118: 155–164.

[65] Field A (2009) Discovering statistics using SPSS. London: SAGE 3rd edition.

[66] Birdsong D (2009) Age and the end state of second language acquisition. In: Ritchie WC, Bhatia TK, editors, The new handbook of second language acquisition, Bingley: Emerlad. 401–424.

[67] Bialystok E (2002) On the reliability of robustness: A reply to DeKeyser. Studies in Second Language Acquisition 24: 481–488.

[68] Rothman J (2008) Why all counter-evidence to the critical period hypothesis in second language acquisition is not equal or problematic. Language and Linguistics Compass 2: 1063–1088.

[69] Nickerson RS (1998) Confirmation bias: A ubiquitous phenomenon in many guises. Review of General Psychology 2: 175–220.

[70] Firebaugh G (2007) Replication data sets and favored-hypothesis bias: Comment on Jeremy Freese (2007) and Gary King (2007). Sociological Methods & Research 36: 200–209.

[71] Fugelsang JA, Stein CB, Green AE, Dunbar KN (2004) Theory and data interactions of the scientific mind: Evidence from the molecular and the cognitive laboratory. Canadian Journal of Experimental Psychology 58: 86–95. 15285598 10.1037/h0085799

[72] Koehler JJ (1993) The inuence of prior beliefs on scientific judgments of evidence quality. Organizational Behavior and Human Decision Processes 56: 28–55.

[73] Mynatt CR, Doherty ME, Tweney RD (1977) Confirmation bias in a simulated research environment: An experimental study of scientific inference. Quarterly Journal of Experimental Psychology 29: 85–95.

[74] Bakker M, Wicherts JM (2011) The (mis)reporting of statistical results in psychology journals. Behavior Research Methods 43: 666–678. 21494917 10.3758/s13428-011-0089-5 PMC3174372

[75] Kroll JF, De Groot AMB, editors (2005) Handbook of bilingualism: Psycholinguistic approaches. New York: Oxford University Press.

[76] Birdsong D, editor (1999) Second language acquisition and the critical period hypothesis. Mahwah, NJ: Lawrence Erlbaum.

