# Correction: The Critical Period Hypothesis in Second Language Acquisition: A Statistical Critique and a Reanalysis

**DOI:** 10.1371/journal.pone.0102922

**Published:** 2014-07-17

**Authors:** The PLOS ONE Staff

Errors occurred during the production of this article in the References section. References 23, 25, 30, 42, and 72 are incorrect. The publisher apologizes for these errors. Please see the corrected version of these references here.

23. Johnson JS, Newport EL (1989) Critical period effects in second language learning: The influence of maturational state on the acquisition of English as a second language. Cognitive Psychology 21: 60–99. doi: 10.1016/0010-0285(89)90003-0

25. Bialystok E, Miller B (1999) The problem of age in second-language acquisition: Influences from language, structure, and task. Bilingualism: Language and Cognition 2: 127–145. doi: 10.1017/s1366728999000231

30. McDonald JL (2000) Grammaticality judgments in a second language: Influences of age of acquisition and native language. Applied Psycholinguistics 21: 395–423. doi: 10.1017/s0142716400003064

42. Simmons JP, Nelson LD, Simonsohn U (2011) False-positive psychology: Undisclosed flexibility in data collection and analysis allows presenting anything as significant. Psychological Science 22: 1359–1366. doi: 10.1177/0956797611417632

72. Koehler JJ (1993) The influence of prior beliefs on scientific judgments of evidence quality. Organizational Behavior and Human Decision Processes 56: 28–55. doi: 10.1006/obhd.1993.1044
